# The clinical features and diagnosis of Metachromatic leukodystrophy: A case series of Iranian Pediatric Patients

**Published:** 2015

**Authors:** Sayena JABBEHDARI, Elham RAHIMIAN, Narjes JAFARI, Sara SANII, Simin KHAYATZADEHKAKHKI, Habibe NEJAD BIGLARI

**Affiliations:** 1 Students’ Research Committee, Faculty of Medicine, Shahid Beheshti University of Medical Sciences, Tehran, Iran; 2 Neuroradiologist, Haghighat Radiology Center, Tehran, Iran; 3 Pediatric Neurologist, Shahid Beheshti University of Medical Sciences, Tehran, Iran; 4 Department of Neonatology, Shahid Beheshti University of Medical Sciences, Tehran, Iran; 5 Pediatric Neurology Research Center, Shahid Beheshti University of Medical Sciences, Tehran, Iran

**Keywords:** Metachromatic leukodystrophy, Neurometabolic disorder, Children

## Abstract

**Objective:**

Metachromatic leukodystrophy disorder (MLD) is one of the rare neurometabolic diseases caused due to lack of saposin B and arylsulfatase A enzyme deficiency.

**Materials & Methods:**

Eighteen patients diagnosed as metachromatic leukodystrophy in the Neurology Department of Mofid Children’s Hospital in Tehran, Iran between 2010 and 2014 were included in our study. The disorder was confirmed by clinical, EMG-NCV, arylsulfatase A enzyme checking and neuroimaging findings along with neurometabolic and genetic assessment from reference laboratory in Iran. We assessed age, gender, past medical history, developmental status, clinical manifestations, and neuroimaging findings of 18 patients with metachromatic leukodystrophy.

**Results:**

From 18 patients, 80% were offspring from consanguineous marriages. A family history of metachromatic leukodystrophy disease was positive for four patients. Twelve patients had late infantile form of this disorder and six patients had juvenile form. A history of tonic type seizure was positive in 20% of the patients and tonic spasm was confirmed with clinical information. Electromyographgraphy (EMG) in 96% of patients was abnormal with demyelinating sensorimotor neuropathy pattern. MRI in all patients showed the leukodystrophic pattern as arcuate fibers sparing and subcortical rim in white matter and periventricular involvement. Our diagnosis was confirmed by EMG-NCV findings with sensorimotor neuropathy pattern and the assessment of arylsulfatase A enzyme function.

**Conclusion:**

MLD is an inheritance metabolic disorder, which was confirmed by the assessment of arylsulfatase A enzyme function, peripheral blood leukocyte that assessed in a referral laboratory in Iran.

## Introduction

Metachromatic leukodystrophy (MLD) is one of the rarest lysosomal disorders, caused because of arylsulfatase A enzyme deficiency. MLD is classified to three types include late infantile, juvenile and adult form (1). MLD is one the diseases, which deteriorate rapidly and occur during the first years of age (2, 3). This disorder can cause the loss of function in cognitive and motor also lead to extensive damage in white matter (4). Signal hyperintensities can be found on brain MRI (5) with white and gray matter involvement. The accumulation of sulfatide in the kidneys, gallbladder and central and peripheral nervous system can cause many organ dysfunctions (6). As a natural course of MLD, the motor deterioration is a key feature (2). In this study, we present 4 years of experience on metachromatic leukodystrophy from the Pediatric Neurology Research Center of Mofid Children’s Hospital, Tehran, Iran. We describe clinical symptoms and neuroimaging findings of 18 cases with this disorder.

## Materials & Methods

**Subjects**

This observational study was conducted in patients diagnosed as metachromatic leukodystrophy at the Neurology Department of Mofid Children’s Hospital in Tehran, Iran (2010–2014). Forty-seven patients (aged from 5 to 96 months) were enrolled in our study with features of neuro-developmental regression and white matter involvement with leukodystrophic pattern in brain MRI. The patients, who in spite of leukodystrophy in their brain MRI had other metabolites increased in their serum tandem mass spectrometry and the ones with high N-acetylaspartate (NAA) acid in their MRS (magnetic resonance spectroscopy) were excluded. Patient information Eighteen of patients were diagnosed as metachromatic leukodystrophy based on neuroimaging findings and decreased function of arylsulfatase A enzyme, assessed in the patients’ peripheral blood leukocytes in a referral laboratory in Tehran, Iran. Data analysis The data were analyzed in this observational study by descriptive methods and no statistical testing was applied.

**Ethical approval**

Institutional ethical approval for the conduct of this study was obtained from the Ethics Committee of Pediatric Neurology Research Center of Shahid Beheshti University of Medical Sciences, Tehran, Iran. All parents signed an informed consent for participation in the study.

**Fig 1:**
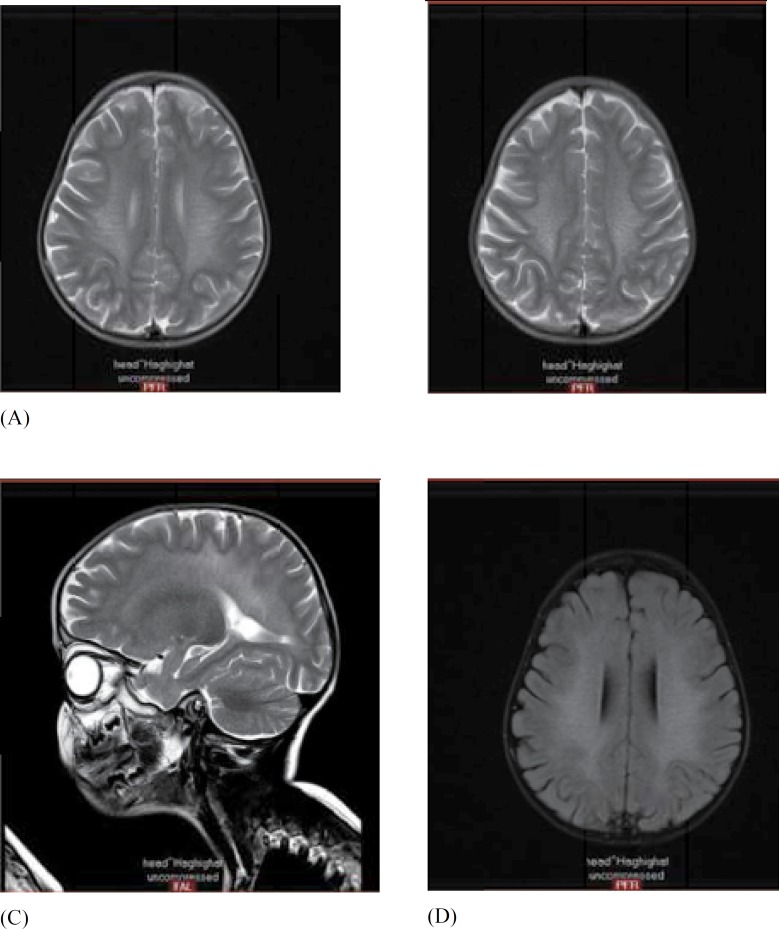
**A) **axial T2WI MR shows the typical butterfly-shaped pattern of white matter involvement in MLD. Note the sparing of internal/external capsules and subcortical U-fibers

**B, C) **Axial and Sag T2WI MR shows confluent, symmetric, hyperintense signal in the white matter. Multiple hypointense lines and dots within the white matter ~ create the characteristic “tigroid” or “leopard” pattern of MLD. The sparing of perivenular myelin is thought to account for this appearance

**D) **Axial FLAIR MR demonstrates the characteristic confluent, symmetric, central white matter involvement of MLD. The “tigroid” or “leopard” pattern of lines and dots within the cerebral hemispheric white matter is evident.

## Results

**Patient information**

Metachromatic leukodystrophy disease was confirmed in 18 patients (10 males and 8 females). The youngest and oldest of all were 18-months and 8 years old, respectively. The median age of both presenting and assessing signs and symptoms (late infantile and juvenile forms) was 42 months. Eighty percent of patients were offspring of consanguineous parents. Forty five percent of them had parents who were first cousins. The resting‘s parents were second cousins. A family history of Metachromatic leukodystrophydisease was positive for four patients.

**Clinical results**

A prenatal history of patients showed a history of preeclampsia in one patient, which leaded to the cesarean section and neonate hospitalized for one day. Prenatal history revealed that two patients had a history of prematurity with gestational ages of 34 and 35 weeks (with normal weight and compatible with gestational age and no history of IUGR); one of them at neonatal period was admitted Neonatal Intensive care Unit because of mild respiratory distress and was discharged without any complication after 3 days. Of 18 patients with metachromatic leukodystrophy, 12 had late infantile form of this disorder, which was begun in 1.5-3 years of age, and they were normal before the diagnosis. The first and the most common complaint among the patients in neurological examination was ataxia, gait disorders and dysarthria. Seven patients with late infantile form of MLD showed cranial nerve impairment such as esotropia and strabismus. The mental and motor regressions process lasted about 10.5 months. A history of tonic type seizure was positive in 20% patients and tonic spasm was seen in 35% patients. Pyramidal involvement included spasticity and increased deep tendon reflex (DTR) seen in 45% of patients, decrease DTR in 35% and dystonia and extarpyramidal involvement in 34% of patients at admission time. All of tonicity was spastic. Ophthalmic and ear examinations showed visual impairment and ear involvement in four patients. After 2 years of having this disorder, swallowing and feeding disorders (Dysphagia) were seen. Among patients with metachromatic leukodystrophy, six had juvenile form of this disorder, which was begun in 3-6.5 years of age; 15% of them had mild speech delay and 12% had motor delay, which had been compensated after 3 years. The patients with juvenile form of MLD showed findings as follows: the onset of symptoms in this group was started with ADHD and irritability; continued with gait impairment and drowsiness as well as gradually dysarthria. Seizure was seen in 25% of patients with dominancy of partial seizure. The progression of juvenile form was slower than late infantile form. Patients with juvenile form of metachromatic leukodystrophy had less cranial involvement and more ataxia than late infantile form. All of the patients had motor involvement and preneural neuropathy, which was one of the best points to be considered as the sign of metachromatic leukodystrophy. The other findings in physical examination were not remarkable.

**Para clinical results**

EMG-NCV in 96% of patients (both juvenile and late infantile form of metachromatic leukodystrophy) was abnormal with demyelinating sensorimotor neuropathy pattern. Visual and auditory involvement in patients with juvenile form of metachromatic leukodystrophy, assessed by physical examination, VEP and ABR were more than late infantile form (70% versus 45%).

**Neuroimaging results**

MRI in all patients showed the leukodystrophic pattern as periventricular white matter involvement with U fiber and subcortical rim sparing (Figure 1). The first and best paraclinical choice for early detection after clinical findings was brain MRI, which showed the leukodystrophic pattern in all of the patients.

## Discussion

Metachromatic leukodystrophy is one of the rare lysosomal diseases. In the late infantile form and juvenile form of this disorder, the motor deterioration is a key feature (7). Lugowska et al. showed that in patients with neuro imaging findings and ataxia, metachromatic leukodystrophy should be considered as diagnosis. Their diagnosis was based on demyelination in brain MRI and detection of low activity of arylsulfatase A (1). Our study showed the prevalence of gait impairment and ataxia in patients with metachromatic leukodystrophy. Wittig et al. reported two cases of infantile MLD in monozygotic twins. They were born from a second cousin marriage (8). In our study, we showed 80% of patients were offspring of consanguineous marriages. Forty five percent of patients had parents who were first cousins and in other patients, their parents were second cousins. It means that in cases with developmental regression and consanguineous marriages the metachromatic leukodystrophy diagnosis should be considered. Prenatal history revealed that two patients had a history of prematurity with gestational ages of 34 and 35 weeks. Congenital anomalies were not detected in any newborn babies at birth. In a study, conducted on brain N-acetylaspartate level in 13 patients with late infantile metachromatic leukodystrophy, the positive association between NAA level, cognitive, and motor function was reported (3). Groeschel et al. showed the decreased amount of gray matter in 18 patients with late infantile MLD (9). In our patients, MRI showed the leukodystrophic pattern as periventricular white matter involvement with U fiber and subcortical rim sparing in all of the patients. Yang et al. showed the rapid and progressive regression of motor development in patients with late infantile MLD (10). Sevin et al. in their review article demonstrated the demyelination as a pathological hallmark in patients with MLD (11). Kehrer et al. in their study done on 59 patients including 27 males and 32 females with MLD (21 late infantile MLD and 38 patients with juvenile MLD) showed all the patients with late infantile MLD had loss of the all gross motor function and patients with juvenile MLD had a more variable motor decline (2). Twelve of 18 patients with metachromatic leukodystrophy in our study had late infantile form of this disorder, which was begun in 1.5-3 years of age, and they were normal before the diagnosis. The progression of juvenile form was slower than late infantile form. Patients with juvenile form of MLD had less cranial involvement and more ataxia than late infantile form. Deconinck et al. presented a case of late infantile MLD disease with normal arylsulfatase A activity (4). Sensorimotor neuropathy pattern and brain imaging findings in our patients lead us to check the arylsulfatase A enzyme function. Eichler et al. reported the confluent T2 hyperintensities of white matter in 33 patients with MLD (10 patients with late infantile, 16 patients with juvenile and 2 patients with adult MLD) (12). Singh et al. showed the isolated cranial neuropathy, spastic gait and decreased level of leukocyte arylsulfatase A activity in a 25-month-old female without intraparenchymal white matter involvement (13). Both the lower and upper motor neuron involvement are seen in patients with metachromatic leukodystrophy; therefore either absence or exaggeration of DTR should not sinister the diagnosis. In conclusion, in patients with developmental regression, ataxia and gait disorder, peripheral neuropathy in NCV, leukodystrophy with specific pattern, positive familial history of MLD and consanguinity marriage of parent, the diagnosis of MLD should be considered.

## Notes

**Funding:** The authors received no financial support for the research and publication of this article.

## Notes

**Declaration of conflicting interests**: None declared.
